# Graphene Decorated Zinc Oxide and Curcumin to Disinfect the Methicillin-Resistant *Staphylococcus aureus*

**DOI:** 10.3390/nano10051004

**Published:** 2020-05-25

**Authors:** Mohammad Oves, Mohd. Ahmar Rauf, Mohammad Omaish Ansari, Aftab Aslam Parwaz Khan, Huda A Qari, Mohamed F. Alajmi, Samaresh Sau, Arun K Iyer

**Affiliations:** 1 Center of Excellence in Environmental Studies, King Abdul Aziz University, Jeddah 21589, Saudi Arabia; 2 Department of Biological Science, Faculty of Science, King Abdulaziz University, Jeddah 21589, Saudi Arabia; dr_hudaqari@hotmail.co.uk; 3 Use-Inspired Biomaterials & Integrated Nano Delivery (U-BiND) Systems Laboratory, Department of Pharmaceutical Sciences, Eugene Applebaum College of Pharmacy and Health Sciences, Wayne State University, Detroit, MI 48201, USA; hb7059@wayne.edu (M.A.R.); gi7517@wayne.edu (S.S.); arun.iyer@wayne.edu (A.K.I.); 4 Center of Nanotechnology, King Abdul Aziz University, Jeddah 21589, Saudi Arabia; omaishchem@gmail.com; 5 Chemistry Department and Center of Excellence for Advanced Materials Research, King Abdul Aziz University, Jeddah 21589, Saudi Arabia; draapk@gmail.com; 6 Department of Pharmacognosy, College of Pharmacy, King Saud University, Riyadh 11451, Saudi Arabia; malajmii@ksu.edu.sa

**Keywords:** GrZnO nanocomposites, antibacterial, anti-biofilm, Graphene, MRSA, Curcumin

## Abstract

Sometimes, life-threatening infections are initiated by the biofilm formation facilitated at the infection site by the drug-resistant bacteria *Staphylococcus aureus*. The aggregation of the same type of bacteria leads to biofilm formation on the delicate tissue, dental plaque, and skin. In the present investigation, a Graphene (Gr)-based nano-formulation containing Curcumin (C.C.M.) and Zinc oxide nanoparticles (ZnO-NPs) showed a wide range of anti-microbial activity against Methicillin-resistant Staphylococcus aureus (MRSA) biofilm and demonstrated the anti-microbial mechanism of action. The anti-microbial effect of GrZnO nanocomposites, i.e., GrZnO-NCs, suggests that the integrated graphene-based nanocomposites effectively suppressed both sensitive as well as MRSA ATCC 43300 and BAA-1708 isolates. The *S. aureus* inhibitory effect of GrZnO-NCs improved >5-fold when combined with C.C.M., and demonstrated a M.I.C. of 31.25 µg/mL contrasting with the GrZnO-NCs or C.C.M. alone having M.I.C. value of 125 µg/mL each. The combination treatment of GrZnO-NCs or C.C.M. inhibited the M.R.S.A. topical dermatitis infection in a mice model with a significant decrease in the CFU count to ~64%. Interestingly, the combination of C.C.M. and GrZnO-NCs damaged the bacterial cell wall structure, resulting in cytoplasm spillage, thereby diminishing their metabolism. Thus, owing to the ease of synthesis and highly efficient anti-microbial properties, the present graphene-based curcumin nano-formulations can cater to a new treatment methodology against M.R.S.A.

## 1. Introduction

The rising case of anti-microbial resistance in bacterial pathogens speaks to a vital risk being posed to human wealth [1,2]. The recently emerging bacterial isolates are resistant to conventional antibiotics attributable owing to factors like uncontrolled antibiotic practice, gene manipulations by the bacterial system, limited diffusion of antibiotics, and development of biofilms, etc. [3,4]. A biofilm is a complex network of microorganisms that irreversibly cling to one another and a substratum, on biotic and abiotic surfaces under several environmental stress conditions, such as the absence of nutrient supplements and oxygen. The biofilm prevents the effect of antibiotics. Staphylococci are perceived as the most frequent cause of biofilm-related infections. The development of biofilm in *S. aureus* infection increases the cost of medical care. Consequently, biofilms are more resistant to most available anti-microbial agents, resulting in treatment failure. There is an essential need for some inventive and naturally well-disposed techniques that can follow in the total elimination of microbial obstruction. In this way, there is a critical requirement in the advancement of some inventive and ecologically agreeable systems that can result in the total killing of microbial resistance.

To this end, metal-based nanomaterial, just as nanoparticle related antibacterial agents, can restrain anti-microbial obstruction [5,6]. The high surface to volume extent, just as extraordinary surface properties of metallic nanoparticles, makes them a robust, adaptable, and compelling anti-microbial therapeutic agent [7,8]. Among different metal oxides, Zinc oxide (ZnO) has been entitled as a generally recognized as safe by the Food and Drug Administration (F.D.A). and engage the status of reliable bactericidal agents [9,10,11]. It is perceived as one of the most active metal-based nanoparticles, as they sharply diffuse into the membrane and damage the microorganism’s cells. Zinc oxide nanoparticles have been utilized as a powerful antibacterial substance against specific pathogens in vitro and in vivo against disease-causing agents. It has been established that most of the metal oxide nanoparticles, including zinc oxide nanoparticles cause injuries to the cell wall and membranes of microorganisms. During the investigations, the electron microscopy studies showed that the zinc oxide nanoparticles on the first contact with the bacteria injure its cell wall. From this injured point, the oxide particles infiltrate, and finally gather inside the cell and on the membrane. They tangle with the metabolic exercises of the microorganism cells, eventually causing death and decay [12,13].

Further, ZnO-NPs may also make an overall impairment of the plasma membrane that eventually leads to cell death promptly. Lamentably, the ZnO-NPs, under specific conditions, eventually lose their capability to disturb the plasma membrane. It is, in this manner, alluring to grow newer ZnO based nano-formulations that show useful antibacterial properties with negligible lethality to the eukaryotic cells.

Graphene Oxide (G.O.) has been accounted for to have astonishing physical and synthetic properties [14,15]. A layer of “sp2” can describe the G.O. hybridized hexagonal carbon sheets with a bunch of hydroxyl and epoxy functionalized groups on their surface and carboxyl gatherings on their edges. The different functional groups of G.O. makes it a perfect framework for its conjugation with metal and metal oxide N.P.s [16,17]. The rhizome of *Curcuma longa* is the scientific name of turmeric, and its vegetative parts contain Curcumin. Curcumin is a phenolic compound, belonging to the class of curcuminoids, with a diarylheptanoid structure. It is widely used for the development of a number of therapeutic medicines [18,19]. Studies have demonstrated that C.C.M. possesses strong anti-microbial potential against a wide range of bacterial and fungal pathogens. The infective counter achievement of C.C.M. can correspond with the modulation of virulence, quorum sensing, as well as biofilm inhibition, etc. [20,21,22]. The previous reports have demonstrated the antibacterial capability of C.C.M. against many drug-resistant strains [23,24,25]. The Curcumin has rich healing properties due to the higher content of bioactive molecules of phenolic compounds, which have powerful anti-microbial properties and also helps in the repression of M.R.S.A. infection. Studies have demonstrated that under in-vitro conditions, the effectiveness of Curcumin is highly enhanced when combined with other antibacterial agents. Thus, keeping in mind the previously mentioned facts, the combination of C.C.M. and GrZnO nanocomposites, i.e., (GrZnO-NCs) have been studied and analyzed for their synergistic anti-microbial impact against two different bacterial strains.

## 2. Materials and Methods

Tryptic Soy Broth (T.S.B.) Culture media and agar were procured from Hi-media (HiMedia Laboratories LLC, Kennett Square, PA, USA). Zinc acetate and graphite powder were purchased from Fischer Scientific (Waltham, MA, USA). Curcumin of 99% purity was purchased from the Sigma Aldrich, St. Louis, MO, USA (Supelco, Bellefonte, PA, USA). The Methicillin-resistant *Staphylococcus aureus* (ATCC BAA-1708 and ATCC 43300) bacterial strains were procured from the A.T.C.C (Manassas, VA, USA). The standard strains were sub-cultured in Tryptic Soy Broth (T.S.B.). The cultures were stored at −20 °C in 20% glycerol for long-term preservation. All experiments were carried out with freshly grown cultures. All other chemicals and solvents used were of analytical grade and acquired locally. Details of the chemicals and animal studies are described in the “Material and Methods” Section of the Supplementary Information (S.I. file). Each animal-related study was executed according to the Guidelines for the Care and Use of Research workplace Animals, as communicated by the Department of Biological Sciences, King Abdulaziz University, and collaboration of Department of Pharmaceutical Sciences, Eugene Applebaum College of Pharmacy and Health Sciences, Wayne State University, Detroit, MI 48201, USA.

### 2.1. Synthesis of Reduced Gr-ZnO Nanocomposite

The Hummer’s and Offemens technique was utilized for the synthesis of Graphene oxide (G.O.), which was obtained from graphite powder with slight modifications [25,26]. The integrated G.O. nanostructure was extensively washed with 4% HCl and finally with water until neutral pH was accomplished. The obtained G.O. nanostructure was then dried in a vacuum oven at 40 °C for 12 h. The modification of GrZnO-NCs can be seen elsewhere [27,28]. Briefly, G.O. powder (~1.0 g) was blended with 100 mL of H_2_O and ultrasonicated for 10 min at 25 °C to obtain a G.O. suspension. To the above G.O. suspension, 1 mM aqueous solution of Zn (AcO)_2_. 2H_2_O was added and mixed for 25 min at 55 °C to facilitate the ion exchange process. NaOH solution was added to this suspension of G.O. and Zn (AcO)_2_, which facilitated the formation of ZnO-NPs. The basic pH permits the fast covering of G.O. functional groups, which have been represented to be ominous for the productive interaction of ZnO-NPs with graphene [29,30]. In the end, NaBH_4_ was added to convert the G.O. into reduced graphene oxide (rGO). The procedure brought about the distribution of ZnO-NPs on rGO. The blend was heated for 60 min at 150 °C under continuous stirring/mixing conditions, after which the mixture was cooled, followed by centrifugation at 25,000 g for 30 min to acquire a final GrZnO nanocomposite. After the centrifugation, the GrZnO-NCs were obtained as a dark-colored powdered suspension at the base of the vial, which was washed with an excess of water and ethanol to remove all the impurities and unreacted precursors. Thus, the obtained GrZnO-NCs were put to dialysis against water to remove unbound zinc ions. Finally, the nanocomposite was solidified via freeze-drying under vacuum at 4 °C till further use.

### 2.2. Characterization of As-Synthesized GrZnO-NCs

The characterization of synthesized material of GrZnO-NCs was done by advanced techniques such as scanning electron microscopy, UV-Visible spectroscopy, X-Ray diffraction, FTIR, and dynamic light scattering (D.L.S.).

#### 2.2.1. UV-Visible Spectroscopy

Ultraviolet-Visible (U.V.) spectrum of as-synthesized GrZnO-NCs was recorded on a double beam spectrophotometer (Shimadzu, Kyoto, Japan) operated at a resolution of 1 nm in the range A_200_–A_800_ nm.

#### 2.2.2. Dynamic Light Scattering

D.L.S. technique was used to determine the hydrodynamic size distribution of the particles. Dynamic light scattering (D.L.S.) measurements were performed on a Malvern Zetasizer Nano-ZS90 (ZEN3590, Malvern, UK).

#### 2.2.3. Electron Microscopy

The size and surface morphology of as-synthesized nanocomposites were characterized, employing T.E.M. and S.E.M. analyses following the method described elsewhere. The sample was prepared by placing a drop of reaction product over the gold-coated negative grid, allowing the solution to evaporate. T.E.M. was performed on a J.E.O.L. model electron microscope. The microscope was operated at an accelerating voltage of 1000 kv. For surface morphological analysis, S.E.M. was performed (JSM67500F, J.E.O.L. model, Tokyo, Japan).

#### 2.2.4. X-ray Diffraction Analysis

XRD analysis of as-synthesized GrZnO-NCs was performed in 2θ range of 20–80° (Rigaku Miniflex II, Tokyo, Japan) with Cu K_α_ radiations (λ = 1.5406 Å) operating at a voltage of 30 kV and current of 15 mA. All the diffraction patterns was recorded as step-scan. Briefly, powdered GrZnO-NCs were mounted followed by fixation of the tube voltage and current, and feeding the following parameters: starting 2-theta angle, step-size (typically 0.005 degrees), count time per step (typically 0.05–1 s), and ending 2-theta angle.

### 2.3. Minimum Inhibitory Concentration (M.I.C.) and Antibacterial Efficacy Determined through Agar Diffusion Assay

Minimum inhibitory concentration (M.I.C.) is the lowest concentration of an anti-microbial agent that may prevent the visible growth of microorganisms after the stipulated time frame. M.I.C. is considered as a significant parameter in diagnostic laboratories to ascertain the sensitivity of a microorganism against a particular anti-microbial agent. The M.I.C. value of as-synthesized GrZnO-NCs was determined by the microdilution method against MRSA ATCC 43,300 and MRSA ATCC BAA-1708 strains following the N.C.C.L.S. mandate. The M.I.C. values were estimated based on viability tests performed in 96-well microdilution plates according to the previously developed protocols [31,32].

### 2.4. The Synergistic Anti-Microbial Effect of C.C.M. Supplemented with GrZnO-NCs Determined by the Agar Diffusion Method

For evaluating the synergistic potential of the GrZnO-NCs with C.C.M., the agar plate was inoculated with MRSA ATCC 43300 and MRSA BAA-1708 strains. Nanocomposites were impregnated on sterile discs, and the dishes were marked cautiously and re-incubated at 37 °C for 24 h. The zone of inhibition was denoted the inhibitory capability of the nanoformulations, and each test was performed in triplicate [31,32].

### 2.5. M.R.S.A. Viability Assay Employing SYTO9 and Propidium Iodide (P.I.) Dyes

To examine the cell viability upon treatment with synthesized materials, GrZnO-NCs, C.C.M., and GrZnO-CCM, the SYTO9-PI bacterial feasibility Kit (Invitrogen, Carlsbad, CA, USA) was employed [33,34]. Point by point data are given in the Supplementary Material.

### 2.6. Anti-Biofilm Activity

The biofilm assay was performed according to the X.T.T. protocol, which has been recently reported by our laboratory [20,35]. Briefly, the fresh biofilms formation in the plate (96 wells) was eroded with the PBS to take out the non-attached bacterial cells. The developing biofilms were treated with varying concentrations of nanoformulations (GrZnO-NCs, C.C.M., and GrZnO-CCM) for 48 h. For more details of the methodology, supplementary files can be accessed.

Further crystal violet staining was accomplished to assess the biofilm formation. After incubation, the biofilm formed culture well was washed multiple times with PBS buffer, and plate moisture was removed by the inverted plate placed on the paper towel. Further, a 0.1% crystal violet solution was added to the dry wells and incubated for 15 min at 25 °C to estimate the thickness of biofilm [11,32].

### 2.7. Bacterial Susceptibility Against As-Synthesized GrZnO-NCs

The efficacy of synthesized nanoformulations and effect on the CFU count and growth curve assay were assessed in-vitro. Briefly, the overnight grown culture of various bacterial strains was sub-distributed into 6 culture tubes (adjusted density to 10^6^–10^7^ cells/mL). Further, 100 µL aliquot of GrZnO-NCs solution from a stock solution of 10mg/mL followed by N.C.s with C.C.M. and C.C.M. alone and final stock of vancomycin solution (100 µg/mL), Negative control (without culture and formulation) and Positive control (culture + 100 µL PBS) were also run simultaneously. After that, 100 µL suspension from each of the treated and control group tubes was plated in triplicate at two different dilutions (1:1, 1:10) on to the T.S.B. Congo red agar plates and incubated further at 37 °C ^13^. After 24 h of incubation at 37 °C, resultant colony forming units (CFU) were counted, averaged, and expressed as log10 CFU/mL and the counts from three independent experiments.

Similarly, for growth kinetics, the cells were grown in the presence of a different combination of GrZnO and C.C.M., and the absorbance was observed at 660 nm at different time intervals.

### 2.8. The Interaction between Bacteria -N.C.s Showed by Electron Microscopy

M.R.S.A. isolate was exposed to different GrZnO-NCs formulations for 60 min, at 37 °C, with constant agitation (at 250 rpm). The cell suspension was washed five times in a modified-TSB medium to remove unbound or loosely associated nanoparticles. The cells (approximately 10^8^ CFU) that interacted with GrZnO-NCs formulations at a stipulated time interval were prepared and imaged using S.E.M. Additionally, and the bacteria were imaged by transmission electron microscopy (T.E.M.) as well. Briefly, the bacteria (~10^9^ CFU) were fixed with 1% glutaraldehyde in PBS and subsequently exposed to 1% osmium tetroxide in water for 24 h each. The sample was transferred onto a 0.22 μm pore size filter (Millipore, Burlington, MA, USA) and substituted with acetone, and subsequently with liquid CO_2_ in a critical point drying apparatus (S.P.I., Millipore, Burlington, MA, USA). Filter paper sections were metalized with gold by sputter coating and imaged with a JEOL JSM-6390 LV [20,35]. 

### 2.9. Determination of Cell Lyses by Protein Leakage

The protein leakage determined the cell lysis from the bacterial cells after the treatment of different GrZnO-NCs combinations. This assay was performed according to the previously reported article elsewhere [36]. Briefly, M.R.S.A. strains 30 mL (∼10^9^ CFU/mL) suspensions in standard saline solution (0.9% NaCl) were mixed and treated with GrZnO-NCs, C.C.M., and GrZnO-CCM at their sub-MIC concentrations separately. An untreated sample was recognized as a control. After the treatments of all mentioned combinations, all cell vials were incubated for 3 h at 37 °C and followed by centrifugation at 10,000× *g* at 4 °C for 10 min. The supernatant obtained was lyophilized (vacuum lyophilization). Finally, the amount of protein in each sample was determined to utilize a B.C.A. based protein analysis assay [36].

### 2.10. Potential of GrZnO-NCs in the Suppression of Experimental M.R.S.A. Skin Infection

The detailed methodology for animal studies and histopathological analysis have been described in the Supplementary Material.

### 2.11. Statistical Analysis

All the experimental data indicated here are represented as mean ± standard deviations employing one way or two-way ANOVA with the help of Graph-Pad Prism version 6.0, Graph-Pad Software Inc. San Diego, CA, USA Significant values are depicted as *** for *p* ≤ 0.001; ** for *p* ≤ 0.01, and * for *p* ≤ 0.05. The differences between the biochemical activities of treated groups were examined by the Student’s *t*-test as significant variables with *p* ≤ 0.05 were measured cautiously.

## 3. Results and Discussion

### 3.1. Characterization of As-Synthesized GrZnO-NCs

**Figure 1:**
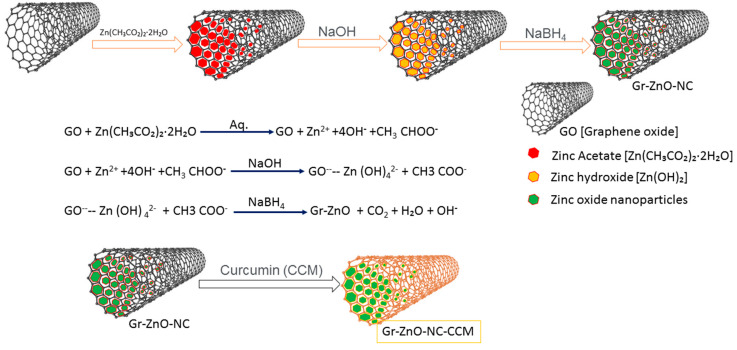
Schematic representation of GrZnO-NCs synthesis by Hummer’s method.

Figure 1 illustrates the efficient fabrication process followed in the synthesis of GrZnO composites. It is well established that the G.O. sheets had carbonyl and carboxyl groups at their edges, while the basal planes of G.O. sheets contain epoxy and hydroxyl groups [37,38,39]. The presence of these functional groups enabled the subsequent in situ attachment of metal nanostructures to the surface as well as at the edges of the G.O. sheets. For example, the addition of 1mM zinc acetate salt to G.O. dispersion allows Zn2+ ions to bind with the O- atoms of the negatively charged residual oxygen-containing functional groups via electrostatic interaction. The addition of 0.1 M NaOH at a relatively higher temperature (approximately 50 °C) led to the formation of a greater number of nuclei. The core Zn atom of the ZnO octahedron forms bond with O atoms of the G.O. functional groups via a covalent coordination bond that may act as an anchor site during the crystal growth. Finally, ZnO particle growth occurs along the surface planes and the edges of G.O. sheets to eventually form G.O./ZnO nanocomposites. Finally, to restore G.O. to sufficiently reduced graphene, reducing agent sodium borohydride (NaBH4) was added. The functional groups on graphene interact with Zn2+ metal ions through electrostatic interaction, thus providing more active growth sites for ZnO-NPs [40,41,42].

**Figure 2:**
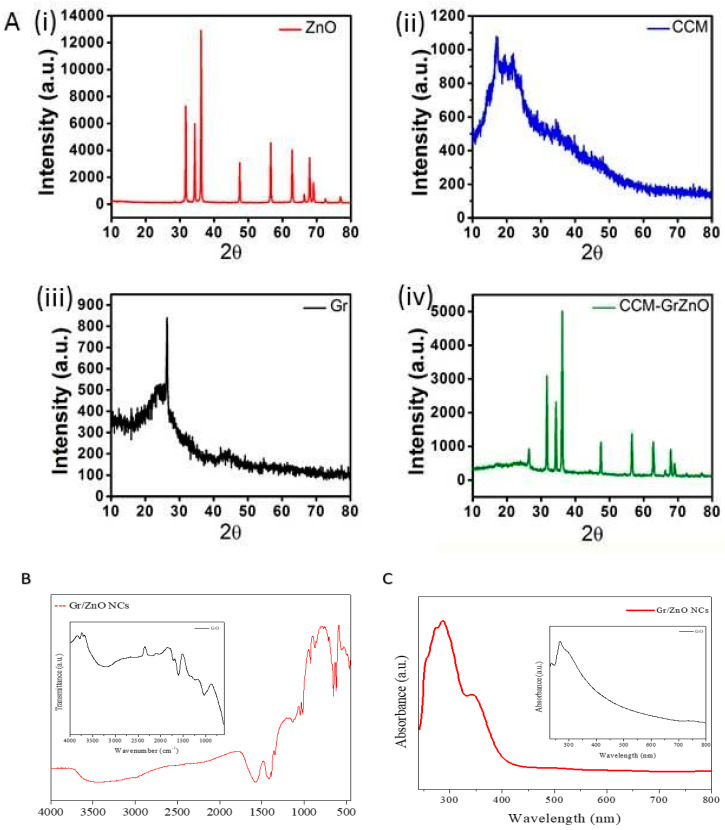
GrZnO-NCs structural characterizations (**A**) XRD patterns of N.P.s were recorded with a 2θ angle in the range of 20–80. XRD pattern of N.C.s showed well-resolved crystalline diffraction peaks. Inset corresponds to the XRD pattern observed for the G.O. sheet. (**B**) Representative FTIR spectrum of the synthesized GrZnO-NCs. The inset represents FTIR for G.O. (**C**) U.V.–visible absorption spectrum of GrZnO-NCs, inset represents spectrum for G.O.

The XRD analysis of ZnO, C.C.M., Gr, and GrZnO-CCM is presented in Figure 2A. Pure ZnO has all the peaks corresponding to its wurtzite structure and is in good agreement with the Joint Committee of Powder Diffraction Standards (J.C.P.D.S.) card No. 36-1451 [43]. C.C.M. showed amorphous regions with few peaks in the region of 15–20 2θ, suggesting its semi-crystallinity. In the case of Gr, a single large diffraction peak at 26.35 2θ correspondings to the (002) plane of the graphite sheet is indicative of the presence of Gr [44]. The final GrZnO-CCM showed all the peaks of ZnO and Gr, thereby suggesting the perfect intercalation of the individual components. The reduction in the overall intensity in the composite can be related to the presence of amorphous C.C.M. and suggests an interaction between Gr, ZnO, and C.C.M. via the ‘π’ electrons of Gr, reactive oxygen site of ZnO and different sites of C.C.M. [39,41,42]. 

The FTIR spectrum of reduced GrZnO-NCs indicated an absorption band at 1601.87 cm^−1^, followed by C=C stretching in the FTIR patterns, which can be correlated with the restoration of the graphene-based C=C network upon reduction. The band observed at 3468.5 cm^−1^ may be assigned to O–H stretching of adsorbed H_2_O molecules. The absorption band at 484.54 cm^−1^ corresponds to the vibration of Zn-O components of the nanocomposite [39,42]. (Figure 2C).

**Figure 3:**
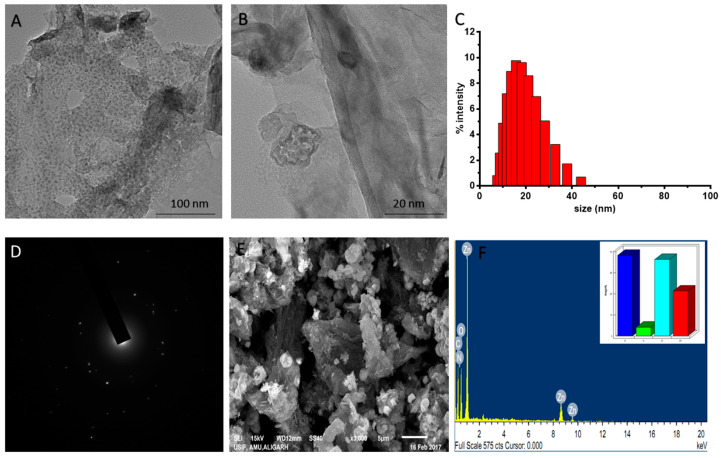
Electron microscopic analysis of as-synthesized GrZnO-NCs. (**A**,**B**). T.E.M. micrograph and (**C**) Size distribution of the synthesized N.C.s (**D**) S.A.E.D. pattern of GrZnO- N.C.s (**E**) S.E.M. micrograph and (**F**) EDX-spectrum representing components of the N.C.s (Inset distribution of components).

The U.V. visible spectra of GrZnO and G.O. (inset) had been depicted in Figure 2C. The synthesized G.O. sample showed an absorption peak at ~221 nm and a shoulder at ~280 nm. The peak at 221 nm can be assigned to the π → π* transition in the aromatic C–C bonds and the shoulder at 280 nm corresponds to n → π* transitions of the C=O bonds [44]. The GrZnO displayed two distinct peaks at ~270 and ~370 nm, respectively, in their U.V. spectrum that may correspond to the excitation of the π- plasmon of both graphitic structures and ZnO-NPs characteristics, respectively. The graphene absorption peak entirely differs from the G.O. peak with redshift to 270 nm indicating a full reduction of graphene oxide to GO-NPs [42,45]. The U.V. absorption data further established the successful synthesis of GrZnO-NCs. The images obtained from the electron microscopic analysis also elucidated the shape and size of reduced GrZnO-NCs. T.E.M. micrographs (Figure 3A,B) of GrZnO-NCs depicted scattered ZnO nanocrystals on the surface of the graphene sheets. The average hydrodynamic size of the synthesized N.C.s was found to be 35 ± 10 nm with a decent polydispersity index (P.D.I.) of 0.313 in a colloidal suspension (Figure 3C). As observed from the S.E.M. image, ZnO-NPs were homogeneously dispersed on graphene sheets (Figure 3E).

Further, E.D.A.X. the test suggested the presence of Zn, C, and O as the most abundant components that represent the purity of the synthesized nanomaterial. The E.D.A.X. spectrum of the nanocomposite additionally affirmed the uniform distribution of Zn and C throughout the sample (Figure 3F). The sporadic spherical surface morphology of ZnO-NPs can be attributed to their impregnation onto the graphene sheet.

### 3.2. The Synergistic Antibacterial Potential of As-Synthesized Reduced GrZnO-NCs in Combination with CCM

**Figure 4:**
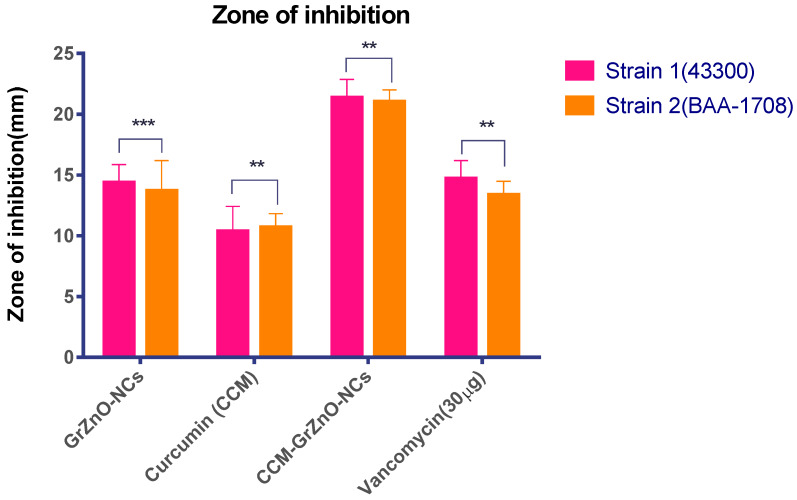
Antibacterial activity of as-synthesized CCM-GrZnO-NCs based formulations. Zone of inhibition as a measure to establish the antibacterial potential of various GrZnO-NCs formulations against M.R.S.A. strains. Experiments were performed in triplicates, and results are shown as mean ± S.D.; ** *p* ≤ 0.01; *** *p* ≤ 0.001

MIC is considered to be an imperative factor in calculating the anti-microbial competency of a drug or a drug candidate against a specific range of pathogens. The lower the M.I.C., the better is the antibacterial is its capacity and vice versa [46]. For our studies, the anti-microbial action of as-synthesized reduced GrZnO-NCs was assessed against the M.R.S.A. isolates. Vancomycin was taken as a control for standard antibiotic The M.I.C. value for the synthesized nanocomposites was around 125 µg/mL for MRSA ATCC 43300 and 250 µg/mL against MRSA ATCC BAA-1708. The C.C.M. alone had a M.I.C. of 125 µg/mL for both the M.R.S.A. strains. Interestingly, GrZnO-NCs and C.C.M. combination displayed the lowest M.I.C. of 31.25 and 62.5 µg/mL for MRSA ATCC 43300 and MRSA BAA 1708 strains, respectively, and our results are consistent with studies performed by previous groups for GrZnO-NCs and Curcumin [47,48,49]. Further, to evaluate the inhibitory potential of as-synthesized nanocomposite and different combinations, Agar diffusion assay was performed (Figure 4).

**Figure 5:**
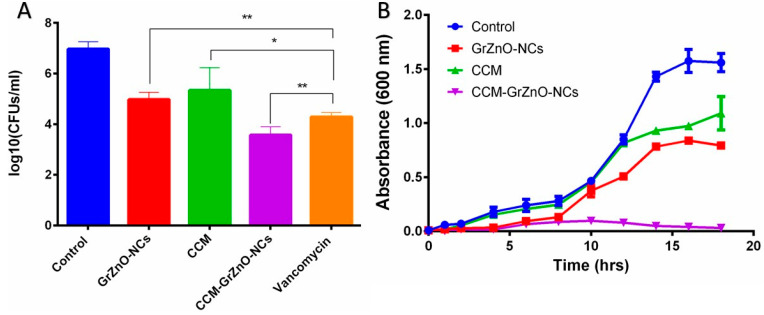
(**A**) Residual bacterial load assay. Bacterial counts (colony forming units) to assess residual MRSA ATCC 43300 survived after exposure to various forms of CCM-GrZnO- N.C.s. Experiments were performed in triplicates, and results are shown as mean ± S.D.; * *p* ≤ 0.01; ** *p* ≤ 0.001. (**B**) Growth kinetics analysis for M.R.S.A. when grown in the presence of different GrZnO-NCs formulations.

The inhibition zone was observed to assess the potent bactericidal activity of nanocomposite against the two tested microorganisms. It seems that the graphene sheet facilitates the deposition of ZnO nanoparticle on the bacterial cell wall, leading to the internalization of zinc oxide nanoparticles that induce R.O.S. production. The R.O.S. generated by internalized ZnO-NPs starts damaging the D.N.A. and other cellular machinery components of the bacteria [11,33]. A much higher M.I.C. and the bactericidal effect was observed for CCM-GrZnO-NCs in contrast to both GrZnO-NCs and C.C.M. This activity occurred due to the slow release of C.C.M. and zinc ions from the combination, which synergistically caused apparent inhibition of both *S. aureus* strains. Next, the SYTO-9 and P.I. dye-based, Live-dead assay also validates the anti-microbial potential of as-synthesized N.C.s against M.R.S.A. strains. Bacterial cells from the log phase were treated with different GrZnO-NCs formulations for three hours, followed by staining with dyes (propidium iodide (P.I.) and SYTO-9 dyes) [50]. In general, P.I. infiltrates cells with disrupted membrane injuries, and the nano-formulation treatment brought about significant staining of cells with P.I. probe. The control group (Live cells) demonstrated green fluorescence while on the other hand, the cells being treated with different nanoformulations for three hours showed brilliant red fluorescence (S.I. file, Figure S1). As expected, the cells after treatment, acquired the P.I. dye and appeared red as sharp red points because of the binding of dye with D.N.A. of killed bacteria. The P.I. binding to the double-stranded D.N.A. is an indicator of N.C. mediated damage of bacterial cell wall [48]. The as-synthesized GrZnO-NCs showed a prominent antibacterial effect against both the M.R.S.A. strains, as revealed by the agar diffusion method (Table 1). The supplementation of GrZnO-NCs with C.C.M. leads to more inhibitory effects as compared to GrZnO-NCs alone. Among various treatment groups, the Gr-ZnO-NC-CCM combination showed profound bacterial inhibition. Next, in-vitro, a CFU count-based antibacterial assay was performed to study the long-term activity of CCM-GrZnO-NCs. The obtained results demonstrated the strong antibacterial potential of GrZnO-NCs alone as well as in combination with C.C.M. (*p*-value < 0.001). The C.C.M. and GrZnO-NCs combination was found to be effective against both M.R.S.A. as well as susceptible to *S. aureus*. Nevertheless, it is worth mentioning that at a given dose set up (1000 µg/mL), the treatment with GrZnO-NCs alone or in combination with C.C.M. showed a significant reduction in CFU when compared to the positive control group (Vancomycin). (Table 2) (Figure 5A). Further, the dynamic effect of GrZnO-NCs and its combinations on bacterial growth were studied. The cells were treated with GrZnO-NCs and C.C.M. combinations at their sub-MIC concentrations, and they exhibited the dynamic behavior against the M.R.S.A. strains compared to the individual treatments in suppressing the growth of bacterial cells (Figure 5B). The extent and amount of cell death increased with an increase in the exposure time. Eventually, the survival capacity of microbes gradually tended to stabilize because of reductions in the density of live bacterial cells.

### 3.3. The Anti-Biofilm Potential of As-Synthesized GrZnO-NCs 

**Figure 6:**
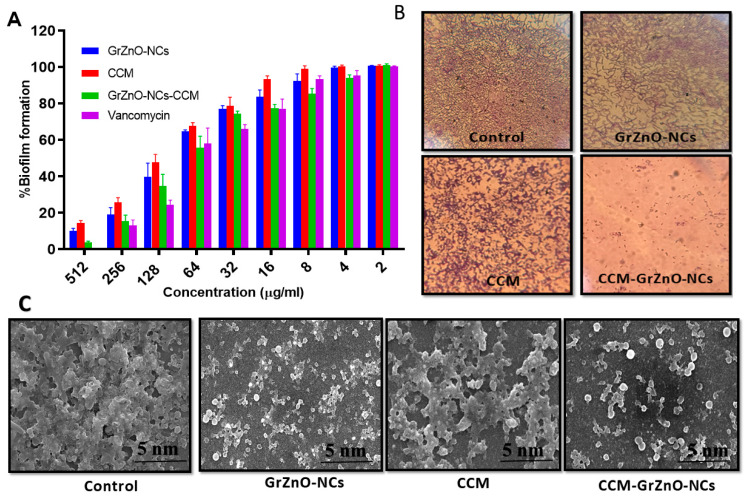
Effect of CCM-GrZnO-NCs various formulations against M.R.S.A. biofilm (**A**). Effect of GrZnO-NCs against biofilm development in MRSA ATCC 43300 strain. Growth inhibition was assessed by comparing relative metabolic activity (R.M.A.) determined using X.T.T. assay; the untreated group served as control showing 100% activity. Experiments were performed in triplicates, and results are shown with mean ± S.D. (**B**) Crystal violet staining of M.R.S.A. biofilm and (**C**) S.E.M. observation of the MRSA ATCC 43300 biofilm upon exposure to GrZnO-NCs formulation.

The unusual activity of an anti-microbial agent is to eradicate bacterial adhesion and prevent the biofilm formation. This activity led to the investigation and application of our synthesized formulation as an anti-biofilm agent, as the bacterial adhesion is considered to be the most crucial stage in biofilm formation [51,52,53]. Thus, to prevent the intractable infections caused by biofilms, the mandatory step is the elimination of adhered bacteria at the early stage of biofilm formation. To examine the anti-biofilm nature of our formulation, the X.T.T. assay and crystal violet staining were performed. The results exhibited the dose-dependent anti-biofilm effect of GrZnO-NCs and CCM-GrZnO-NCs. Further, C.C.M. supplemented GrZnO-NCs inhibited the biofilm more efficiently as compared to monotherapy (individual treatment) with GrZnO-NCs and C.C.M. alone, as evident from X.T.T. assay and crystal violet staining assay (Figure 6A,B). The presence of C.C.M. allows the slower release of zinc ions. The prolonged-release of zinc contributes to the antibacterial effect for a more extended period. The enhanced activity can be correlated with the shielding effect of the GrZnO-NCs. Further, S.E.M. observation of the MRSA ATCC 43300 biofilm upon exposure to GrZnO-NCs formulation is depicted in Figure 6C.

On the other hand, C.C.M. seems to exert its anti-biofilm activity via the attenuation of virulence factors, as revealed by the signal molecule-based Q.S. system. The pathogenicity of *S. aureus* involves the synchronized appearance of virulence factors. The agar quorum-sensing framework, which facilitates and enables the pathogen to detect both the density of the nearby *S. aureus* masses and degree of confinement, plays a central role in the regulation of the *S. aureus* virulence. Studies showed that inactivating the agar quorum sensing through small molecules like Curcumin, diallyl sulfide, and quercetin ensues in the inhibition of biofilm formation [22,52,53].

**Figure 7:**
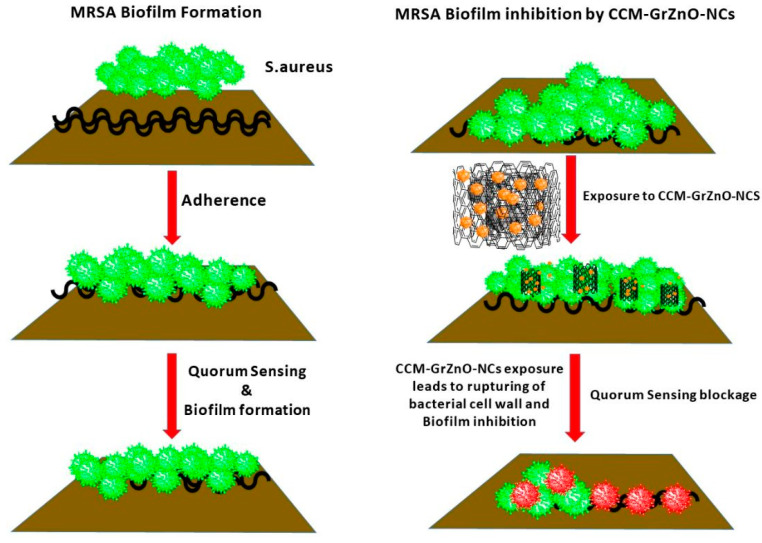
Schematic representation of the proposed mechanism of GrZnO-CCM combination mediated biofilm inhibition and blocking of quorum sensing.

The studies performed envisioned that the combination therapy employed in the present research enhances the anti-microbial as well as the antibiofilm activity of GrZnO-NCs and C.C.M. complex (Figure 7).

### 3.4. Influence of CCM-GrZnO-NCs Formulation on Bacteria as Depicted by E.M. Examination 

**Figure 8:**
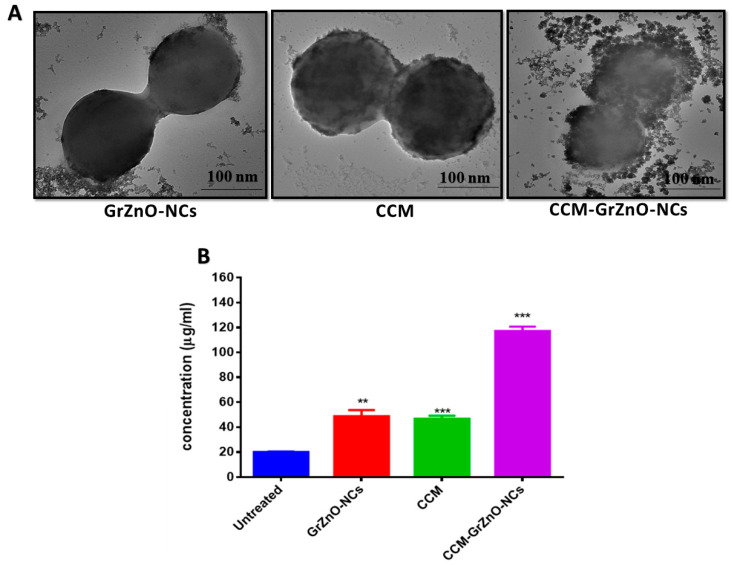
The effect of co-incubation of M.R.S.A. with various GrZnO-NCs. (**A**) T.E.M. was showing the interaction of MRSA ATCC 43300 strain with various GrZnO-NCs and (**B**) Protein leakage from the bacterial cytoplasm. Results are shown as mean ± SD; ** *p* ≤ 0.01; *** *p* ≤ 0.001.

The morphological changes in bacterial cells upon exposure to CCM-GrZnO- N.C.s were deduced employing electron microscopic studies. The control MRSA ATCC 43300 bacterium appeared round with smooth morphological features. The treatment with CCM-GrZnO-NCs formulation resulted in disrupted morphology and damaged surface (Supplementary Figure S2. Thus, it can be interpreted that on treatment with CCM-GrZnO-NCs formulation, the bacterial cell lysis occurs, and consequently, the release of cytosolic content occurs (Figure 8B) [54]. The cytoplasmic leakage owing to the formulation of CCM-GrZnO-N.C.s was further established through quantitative detection of protein leakage, which reflected the appropriate indicator of cytoplasmic leakage. For MRSA ATCC BAA-1708, as shown in Figure 8B protein leakage in the negative control (without treatment) is around 19.2 μg/mL, while GrZnO-NCs and C.C.M. showed 52.2 and 48.6 μg/mL protein leakage, respectively. In contrast, the combinational therapy (GrZnO-CCM) showed 114.8μg/mL protein leakage, which is approximately six times to that of the control and thrice that of the independent groups. These results of protein leakage assay are concurrent with the results obtained from S.E.M. and T.E.M. analysis, thereby signifying that the GrZnO-CCM combination did critical physical damage to the bacterial cell integrity.

**Figure 9:**
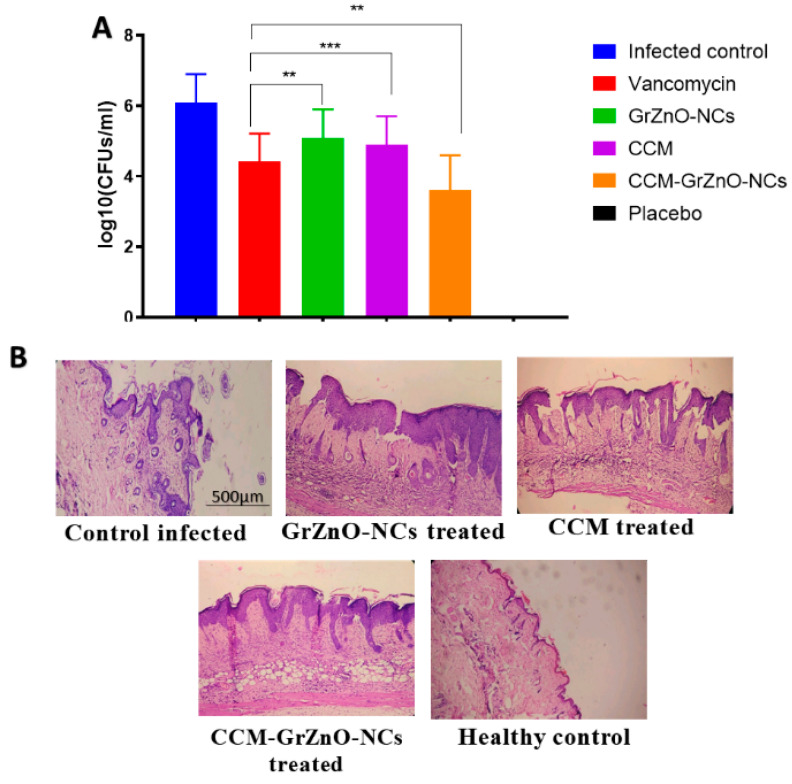
Effect of CCM-GrZnO-NCs formulations on experimental M.R.S.A. skin infection in Balb/C mice. (**A**) Efficacy of CCM-GrZnO-NCs against M.R.S.A. skin infection in experimental animals. Mice were infected topically with MRSA ATCC 43,300 and subsequently treated with various CCM-GrZnO-NCs groups. Mice inoculated with PBS alone served as a control group. After treatment with various GrZnO-NCs, skin lesions were cut, the homogenized and bacterial count was determined by CFU assay on 10th-day post-infection. Results are shown as mean ± SD; ** *p* ≤ 0.01; *** *p* ≤ 0.001. (**B**) biopsy specimens were removed aseptically on 10thday post-infection and fixed in 4% neutral buffered formalin and embedded in parafilm. The biopsy specimens were stained with hematoxylin and eosin before their examination.

Antibacterial potential of CCM-GrZnO-NCs combination against M.R.S.A. induced skin infection in mice models. Keeping into consideration the strong antibacterial potential of GrZnO-NCs against both MRSA ATCC 43300 as well as ATCC BAA-1708 isolates under in vitro conditions. Next, we evaluated its capability to treat acute dermatitis in an animal model. The exposure of Balb/C mice with M.R.S.A. brought about cutaneous infections that were described by reddening of the skin that eventually causes localized skin disruption (Supplementary Figure S3). The residual bacterial burden in the skin was assessed by the enumeration of bacteria existent in the given specimen by culturing it in a solid agar medium. GrZnO-NCs treatment successfully eliminated skin infection (Figure 9A). Improved GrZnO-NCs treatment with C.C.M. brought about a tremendous increment in its capability to decrease the bacterial burden (~64% decrease) when contrasted with untreated control (*p* < 0.005).

### 3.5. Histopathological Analysis

To evaluate the efficacy of different GrZnO-NCs combinations, the skin’s structural design, bacterial load, and acquired inflammatory changes in the mice model were investigated. Ten days post-infection, the treated skin samples were taken out aseptically and subsequently stained by employing hematoxylin and eosin (HE) staining. The lively group exhibited conventional skin histology with a standard unblemished epidermal layer. In contrast, M.R.S.A. infected skin indicated thinning of the epidermal layer with interruption along with a substantial number of inflammatory cells. Following treatment with CCM-GrZnO-NCs based combination, they have brought about the recuperating of the epidermal layer with minimal skin harm. Overall, the new skin attained the architecture similar to more or less healthy skin structure upon therapy with CCM-GrZnO-NCs treatment (Figure 9B).

## 4. Conclusions

Finally, to conclude our study, herein, we have synthesized GrZnO-NCs employing a modified Hummer’s method. The as-synthesized reduced GrZnO-NCs were characterized by applying advanced instrumental techniques viz. U.V. and FT-IR spectroscopy. The surface structure, size dimensions, and crystalline nature were assessed through D.L.S., XRD, and electron microscopy. The GrZnO-NCs showed moderate anti-microbial activity against both resistant as well as sensitive *S. aureus* strains. When the GrZnO-NCs were supplemented with Curcumin (C.C.M.), their anti-microbial and the antibiofilm potential enhanced drastically. The as-synthesized GrZnO-NCs-CCM combination exhibited the substantial potential to suppress the growth of both sensitive and resistant *S. aureus* pathogens. The combinational treatment was additionally fruitful in the repression of *S. aureus*-based skin infection in B.A.L.B./c mice models. From this proof of concept study, it can be concluded that the GrZnO-NCs and their combination with Curcumin (C.C.M.) can act as a potent future anti-microbial nanomaterial for combating biofilms of antibiotic-resistant pathogens.

## Figures and Tables

**Table 1:** Effect of the combination of C.C.M. and GrZnO-NCs against M.R.S.A. strains as revealed by a zone of inhibition assay determined using agar diffusion method. Zone of inhibition observed (in mm).

Strains	GrZnO-NCs	CCM	CCM-GrZnO-NCs	Van (30 µg/disk)
ATCC 43300	14.6 ± 2	10.33 ± 2.082	21.33 ± 2.517	14.333 ± 2
ATCC, BAA-1708	13.3 ± 1.5	9 ± 2	18.33 ± 0.677	9.67 ± 2.5

**Table 2:** Bacterial load in terms of log10 CFU/mL to assess residual MRSA ATCC 43300 survived in response to treatment with various GrZnO-NCs based formulations.

Pathogen	Groups	Log_10_ CFU/mL
**MRSA 43300**	Positive Control	6.17
	GrZnO-NCs	4.393
	CCM	4.773
	CCM-GrZnO-NCs	3.3
	Vancomycin	4.17

## Supplementary Materials

The following are available online at https://www.mdpi.com/2079-4991/10/5/1004/s1, Figure S1: Antibacterial activity of as-synthesized CCM-GrZnO-NCs based formulations. Fluorescence microscopic images are showing MRSA ATCC 43300 cells upon their treatment with various CCM-GrZnO-NCs based formulations, Figure S2. The effect of co-incubation of M.R.S.A. with various GrZnO-NCs. (A) SEM was showing the interaction of MRSA ATCC 43300 strain with various GrZnO-NCs and change in the morphology of the MRSA strains after interaction to different GrZnO-NCs formulations, Figure S3. Effect of CCM-GrZnO-NCs formulations on experimental M.R.S.A. skin infection in Balb/C mice. Efficacy of CCM-GrZnO-NCs against M.R.S.A. skin infection in experimental animals.
