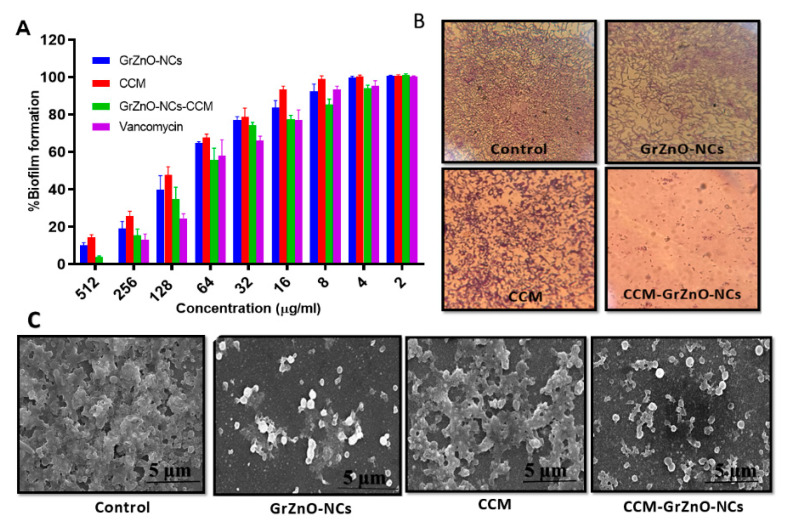# Correction: Oves M, et al. Graphene Decorated Zinc Oxide and Curcumin to Disinfect the Methicillin-Resistant *Staphylococcus aureus. Nanomaterials* 2020, *10*(5), 1004

**DOI:** 10.3390/nano10081453

**Published:** 2020-07-24

**Authors:** Mohammad Oves, Mohd. Ahmar Rauf, Mohammad Omaish Ansari, Aftab Aslam Parwaz Khan, Huda A. Qari, Mohamed F. Alajmi, Samaresh Sau, Arun K. Iyer

**Affiliations:** 1 Center of Excellence in Environmental Studies, King Abdul Aziz University, Jeddah, K.S.A. 21589, Saudi Arabia; 2 Department of Biological Science, Faculty of Science, King Abdulaziz University, Jeddah, K.S.A. 21589, Saudi Arabia; dr_hudaqari@hotmail.co.uk; 3 Use-inspired Biomaterials & Integrated Nano Delivery (U-BiND) Systems Laboratory, Department of Pharmaceutical Sciences, Eugene Applebaum College of Pharmacy and Health Sciences, Wayne State University, Detroit, MI 48201, USA; hb7059@wayne.edu (M.A.R.); gi7517@wayne.edu (S.S.); arun.iyer@wayne.edu (A.K.I.); 4 Center of Nanotechnology, King Abdul Aziz University, Jeddah, K.S.A. 21589, Saudi Arabia; omaishchem@gmail.com; 5 Chemistry Department and Center of Excellence for Advanced Materials Research,King Abdul Aziz University, Jeddah 21589, Saudi Arabia; draapk@gmail.com; 6 Department of Pharmacognosy, College of Pharmacy, King Saud University, Riyadh 11451, Saudi Arabia; malajmii@ksu.edu.sa

The authors wish to make the following corrections to this paper [1]:

There are two mistakes in this article. In **Figure 6C**, the scanning electron microscope images of MRSA in should be in **micrometre** scale—previously, they were shown in **nanometer** scale—and the image in **Subsection B (Figure 6C)** should be at a higher resolution; previously, it was at a different resolution.

The authors would like to **express regret** for any inconvenience caused to the readers by these changes.

**Change in Figure**

The author wishes to make the following correction to this paper [1]. Due to mislabeling, replace


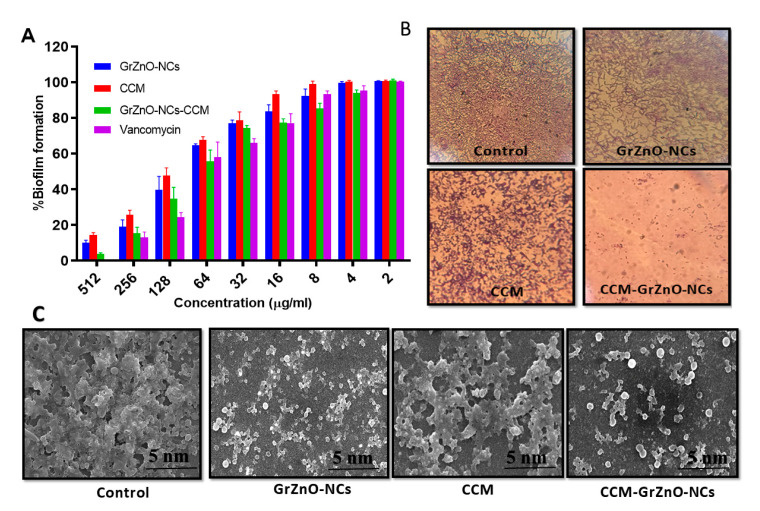
with