# Supplementary material for: Graphene Decorated Zinc Oxide and Curcumin to Disinfect the Methicillin-Resistant Staphylococcus aureus
Source: Nanomaterials (Basel). 2020 May 25;10(5):1004. doi: 10.3390/nano10051004 (PMC7281119; doi:10.3390/nano10051004)
Supplement: Supplementary file 1 [file nanomaterials-10-01004-s001.pdf]

Anti-biofilm Activity

The anti-biofilm nature of synthesized nanocomposites and their combination with CCM were established employing XTT assay against the 2 MRSA strains. Briefly, after the formation of biofilms, the plate was carefully rinsed with sterile PBS to remove non-adherent cells. Afterward, the mature biofilm was incubated with increasing concentration of different GrZnO and CCM nanoformulations and incubated at 37 ºC for 48 hrs. After stipulated time period, 2,3-Bis(2-methoxy-4-nitro-5-sulfophenyl)-5-([phenylamino] carbonyl)-2H-tetrazolium hydroxide (XTT), was added at a final concentration of 5 mg/ml. The above-obtained solution was passed through 0.22-mm pore-size filter and stored at −80°C till use. 0.4mM of Menadione solution was primed and filtered immediately just before the commencement of assay. Adherent bacterial cells were washed with PBS (200 μL), followed by addition of XTT, and two μl of menadione to each well. The solution was transferred to a new plate after incubation in the dark for four hrs at 37°C, and the colorimetric change in the solution was assessed using a microtitre plate reader (BIORAD Microplate reader at 490 nm)[1]. Experiments were performed in triplicate. The data were expressed as mean ± SD.

MRSA viability assay employing SYTO9 and propidium iodide (PI) dyes

To study the cell viability upon treatment with GrZnO-NCs and CCM combination the SYTO9-PI bacterial viability kit (Invitrogen, CA) was used. An aliquot (10μL) comprising of an equal proportion of SYTO9 and PI mixture was added to the treated cells in the six well-plates and incubated for 15 min in the dark at room temperature. After the stipulated incubation time, the cells were washed with cold, sterile PBS and mounted on a glass slide. The cells were seen under a fluorescence microscope(Zeiss, USA) [2,3].

Invivo animal infection

Six to eight-week-old male BALB/c mice were used in the study. Five different groups were organized, having six animals in each group. Firstly, animals from each group were anesthetized by intraperitoneal (IP) injection of a ketamine-xylazine cocktail and subsequently shaved on the dorsal surface. The skin of the mouse was rasped with sterile scalpel blades until a reddened area appeared. The surface of each wound was inoculated with 50 μl of the culture suspension corresponding to 10^8 CFU of MRSA ATCC BAA 1708 strain. The infection was allowed to establish for 96 h. The treatment schedule was followed as:

1. Group 1: Healthy mice exposed to normal saline only.
2. Group 2: Mice exposed to MRSA ATCC BAA 1708, followed by treatment with normal saline only.
3. Group 3: Mice exposed to MRSA ATCC BAA 1708 followed by treatment with GrZnO-NCs (1 g/kg body weight)

4. Group 4: Mice exposed to MRSA ATCC BAA 1708 followed by treatment with CCM (1 g/kg body weight).

5. Mice exposed to MRSA ATCC BAA 1708 followed by treatment with GrZnO-CCM (0.5 g/kg body weight).

Each mouse was equipped with an Elizabethan collar (Braintree Scientific, Braintree, MA) to avert evacuation and ingestion of applied formulations, before the treatment with nanocomposite suspension following the protocol of luke Mortenson et al. 2014. The different grouped animals were treated with several formulations on 3rd, 7th, and 10th day of the experiment after the establishment of the infection. Ten days post-infection; anesthetized mice were sacrificed, and their infected skin part was removed with surgical scissors and homogenized using a tissue homogenizer (Silverson Machines, East Longmeadow, MA, USA), and the residual bacterial burden was determined by plating serially diluted homogenized samples on BHI agar plates [4,5].

For histopathological examination, the skin tissues were taken out from the mice belonging to the different treatment groups followed by fixing in 10% formaldehyde, got dried out in variable concentrations of ethyl alcohol then, cleared in xylol and mounted in molten paraplast at 58– 62 °C. The finely cut thin segments were stained with hematoxylin and eosin dyes and assessed for any morphological changes under an Olympus BX40 magnifying lens (PA, USA) for the relative investigation [3,5].

Ethical conduct of research

In this investigation, ingrained BALB/c mice (6– 2 months old, 20 ± 2 g) were supplied from the Institute's Animal House Facility. All mice were housed in polypropylene confines and kept up ideal temperature conditions on a 12hr light-dark cycle and had free access to food and water. Every animal-based trial was performed by the rules of the National Regulatory Committee for Control and Supervision of Experiments on Animals (CPCSEA), and all the studies were specifically approved by the ethics committee. Our research institute endorsement ID is 332/CPCSEA for the department of Biochemistry (JNMC unit).

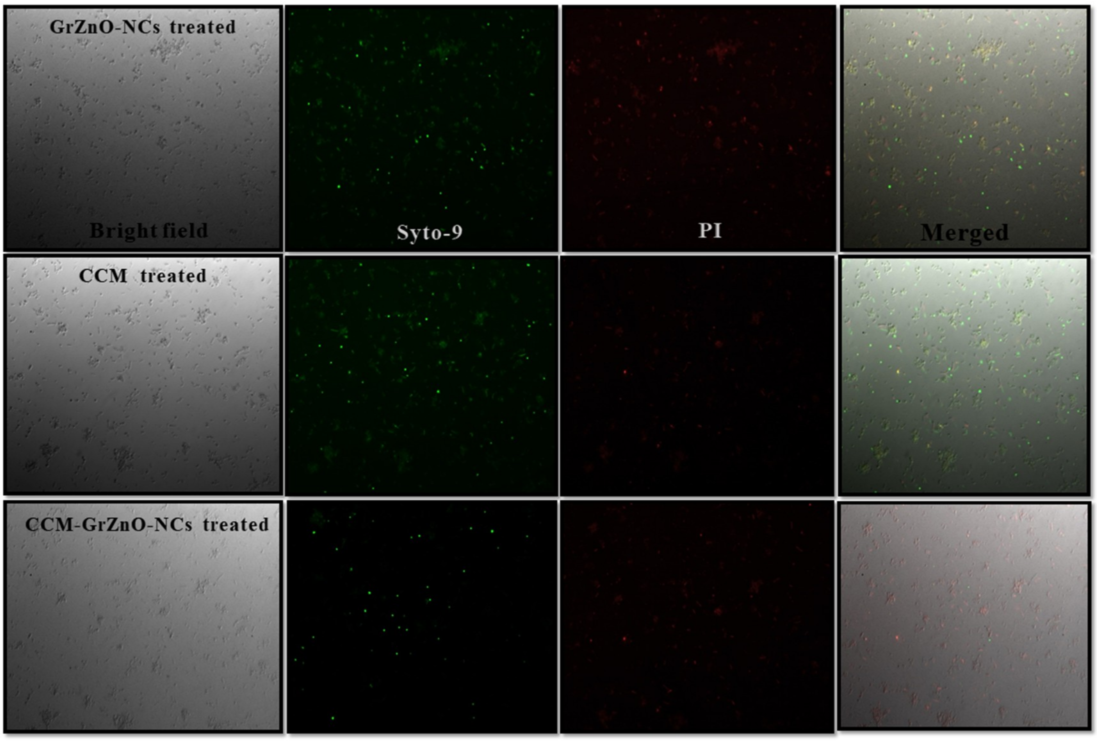

Figure S1: Antibacterial **activity of as-synthesized CCM-GrZnO-NCs based formulations.** Fluorescence microscopic images are showing MRSA ATCC 43300 cells upon their treatment with various CCM-GrZnO-NCs based formulations.

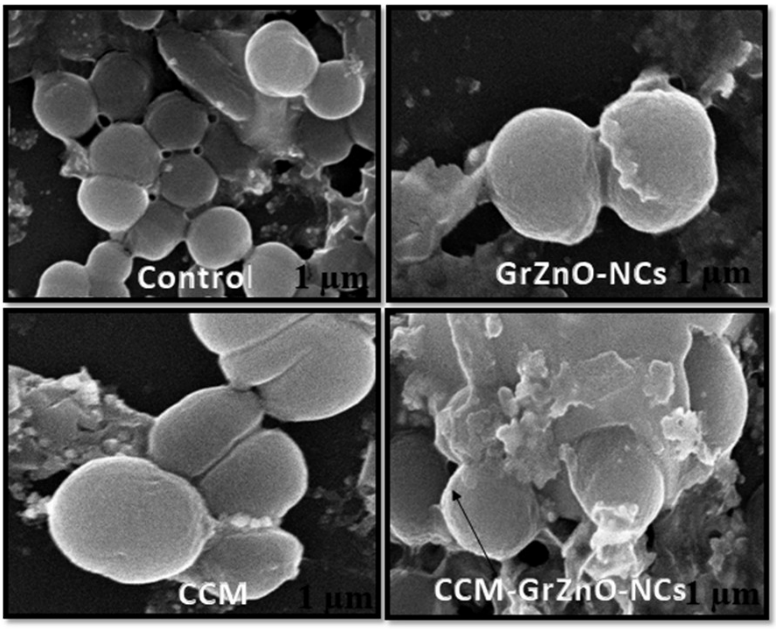

Figure S2. The effect of co-incubation of M.R.S.A. with various GrZnO-NCs. (A) SEM was showing the interaction of MRSA ATCC 43300 strain with various GrZnO-NCs and change in the morphology of the MRSA strains after interaction to different GrZnO-NCs formulations.

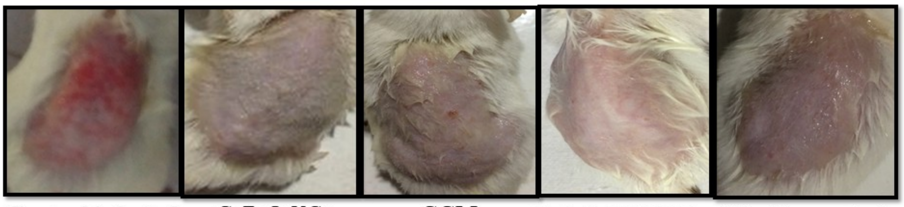

Figure S3. Effect of CCM-GrZnO-NCs formulations on experimental M.R.S.A. skin infection in Balb/C mice. Efficacy of CCM-GrZnO-NCs against M.R.S.A. skin infection in experimental animals.

REFERENCES

1. Ansari A, Ali A, Asif M, Rauf MA, Owais M, Shamsuzzaman. Facile one-pot multicomponent synthesis and molecular docking studies of steroidal oxazole/thiazole derivatives with effective antimicrobial, antibiofilm and hemolytic properties. *Steroids*. 2018;134:22-36. doi:https://doi.org/10.1016/j.steroids.2018.04.003
2. Wiegand I, Hilpert K, Hancock REW. Agar and broth dilution methods to determine the minimal inhibitory concentration (MIC) of antimicrobial substances. *Nat Protoc*. 2008;3(2):163-175.
3. Zhao R, Kong W, Sun M, et al. Highly Stable Graphene-Based Nanocomposite (GO–PEI–Ag) with Broad-Spectrum, Long-Term Antimicrobial Activity and Antibiofilm Effects. *ACS Appl Mater Interfaces*. 2018;10(21):17617-17629. doi:10.1021/acsami.8b03185
4. Rauf, M. A., Owais, M., Rajpoot, R., Ahmad, F., Khan, N., and Zubair, S. (2017). Biomimetically synthesized ZnO nanoparticles attain potent antibacterial activity against less susceptible S. aureus skin infection in experimental animals. *RSC Adv.* 7, 36361–36373.
5. Wu, J., Liu, W., Xue, C., Zhou, S., Lan, F., Bi, L., et al. (2009). Toxicity and penetration of TiO2 nanoparticles in hairless mice and porcine skin after subchronic dermal exposure. *Toxicol. Lett.* 191, 1–8.
